# Epistemic disobedience–Undoing coloniality in global health research

**DOI:** 10.1371/journal.pgph.0003033

**Published:** 2024-04-22

**Authors:** Thirusha Naidu

**Affiliations:** 1 Department of Behavioural Medicine, Nelson R. Mandela School of Medicine University of KwaZulu-Natal, Durban, South Africa; 2 Department of Public Health and Primary Care, University of Cambridge, Cambridge, United Kingdom; University of Sydney, AUSTRALIA

*For the sake of one’s children, in order to minimize the bill that they must pay, one must be careful not to take refuge in any delusion—and the value placed on the color of the skin is always and everywhere and forever a delusion*.

***James Baldwin [1963]. The Fire Next Time**. [1]*

Global health research takes refuge in the delusion that the value placed on Global North and Global South welfare is equal. Funding priorities, research agendas, methodology, research roles, outcomes and dissemination are based on Global North priorities and designed to reinforce Global North superiority. Coloniality in knowledge production is a problem inherited from when the colonial project took control of land, labour, and resources while enforcing the colonisers’ ways of living and thinking [2, 3]. Colonisers delineated criteria for being human in the colonisers’ own image–white, male, heterosexual, able-bodied and cisgender–placing the performance of that humanity at the zero-point, as the original and only legitimate perspective [2].

The colonial project is ubiquitous in the modern world as it is preserved by settler colonial governments [4]. Previously colonised peoples consciously and unconsciously replicate colonialism through popular culture, education and knowledge production, albeit behind a façade of progress or modernity [5, 6]. Colonisation was achieved through physical violence for resource extraction, via the eradication of colonial subjects’ ways of being and thinking [7]. Epistemic violence continues where knowledge systems are overwhelmingly dominated by Global North epistemology. Colonially enforced language and cultural norms still deprive women, people of colour and Indigenous researchers and communities from fully engaging in global health research. Colonially influenced education, resources, and research infrastructure produced the modern Global North-South disparities in research capacity. Global North researchers successfully publish in international journals through access to social capital, research agendas and funding opportunities. People from previously colonised regions are underrepresented in leadership roles in global health research, leading to weak Global South influence and participation in research.

*…I know that what I am asking is impossible. But in our time, as in every time, the impossible is the least that one can demand*.

***James Baldwin [1963]. The Fire Next Time**. [1]*

Epistemic disobedience demands the speciously impossible; provoking violent responses, fragility or denial in those who benefitted, over generations, from current dominant systems. Privilege is legacy-bound. A person’s privileged status must be assessed according to the relative access their ancestors and descendants had to the same resources. Privilege is not attained in the course of a single lifetime. It is a social benefit developed and inherited over generations. Black and Indigenous people have only recently had access to social resources and beneficial social determinants rendering their social and epistemic legacies more precarious than their settler colonial and white counterparts. White saviourism in academia is counterproductive to the decolonial project. It elevates Global North academics as universal knowers and Global South researchers as constrained to learn the system without ever belonging to it.

Practices that teach Global South authors how to write; that make accommodations for submissions from Global South authors to be published; that create special journal editions for Global South work and special regional editions of journals undermine the levelling of Global South and Global North epistemologies. These strategies create temporary gaps in exclusionary systems instead of dismantling them.

Instead reflexivity and positionality statements in published works may initiate a turning point towards dismantling dominant epistemic foundations (Table 1). These statements should be structured to make explicit the different challenges in Global North and Global South researchers’ experiences in the research process as they consider whether they will comply with coloniality in academia or practice epistemic disobedience. Arguably, the closer researchers’ personal and historical identities and research approaches are to dominant views the less likely they are to need to practice epistemic disobedience.

**Table 1:** What are positionality and reflexivity statements?

Positionality statements	Reflexivity statements
Elucidate the researcher’s personal identity relative to the identities of those in the research contexts. Such statements should reveal relative histories and privileges where privilege is inherited by way of personal identity and history.	Describe researchers’ reflections on how their positionality impacted the research relationships, process and outputs. Such statements should reveal the researcher’s reflections in their personal and professional identity relative to research participants’ and the research context and process.

Epistemic disobedience demands that Global South researchers reject oppressive and exclusionary knowledge production machinery [8]. Funding calls obliging Global South researchers to collaborate with Global North researchers to access large grants are blatantly inequitable, representing modern iterations of colonial knowledge and resource extraction and suppressing Global South researchers’ agency. Grants compelling support for Global North students and administration costs for Global North universities over local students and senior researchers in Global South locations, school emerging Global North researchers in modern methods of knowledge appropriation. In projects of Global South interest, Global South students should be given priority over Global North students for funding, training, travel and dissemination. Principal investigators from the Global South must lead Global South -located studies. Beyond acting as reviewers for equity, diversity and inclusion and Global South-related submissions, in journals, Global South scholars must hold influential gatekeeping roles which shape knowledge production narratives e.g as editors and editorial board members. Journal special editions and regional versions dedicated to Global South matters exoticizes knowledge not grounded in the zero-point perspective [9].

*“leave methods to the botanists and mathematicians…build the world of you.”*

***Frantz Fanon [1952]*. *Black Skin*, *White Masks*.** *[6]*

In field, laboratory and practice-based research, epistemic disobedience calls for ‘dereliction of methods’ [10, 11]. Fanon yearned for a decolonial attitude which would challenge established research methods, study populations contexts and topics to construct colonised peoples’ identity beyond the colonial zero-point [6]. Research outputs must transcend funder reports and academic papers. Outputs that communicate results in accessible and meaningful ways include theatre, poetry, videos, and comics serving to deflect epistemic dominance. Transgressive and disruptive ideas should be introduced and debated in commentaries, essays, letters and perspective papers to explain the epistemological turn towards the Global South. Conceptual and dialogical qualitative research must be held in equal esteem to quantitative research. Studying research politics, methods, and the relational and global power dynamics in research teams can reveal the microprocesses that ensure that coloniality and Global North epistemic dominance is still evident in global scientific research.

In scholarship and publication, Global South researchers must insist on significant and senior authorship positions [10, 11]. Prominent author positions legitimise Global North authority in Global South contexts while placating Global South researchers with publications, yet maintain their invisible status. Calls for ‘fair’ authorship allocation in Global Health Research are increasing [12]. Equity over fairness is imperative. Fairness dictates that authorship is considered on academic contribution, regardless of positionality, context and privilege. Equity seeks to level historical and contextual disadvantage by allocating authorship, which elevates voices of historically disadvantaged researchers in neglected contexts. Journals must invite submissions in the regional languages of locations where research is conducted. Multilingual abstracts should be routinely available in prominent global journals. As a global language, variations in English expression and writing style should be expected and supported [13, 14].

Career advancement linked to visibility in publications considered since colonial times persists today. Publication as crucial for visibility is indisputable under current conditions. However, where research is published may not matter as much as *when* new ideas are published. For example, Eve Tuck and Wayne Yang’s article, *“Decolonisation is not a metaphor”*, was published in volume one of a niche journal, and has been cited over 8500 times [15]. This paper drew wide attention because it was published at a time in the discourse on decolonisation; when people were listening in wait for eloquent, emphatic writing about what they knew intuitively and had experienced first-hand. Citation is an underestimated but powerful tool for epistemic disobedience. Researchers can unsettle dominant research narratives by citing the controversial ideas of marginal or diverse authors. Thus, thickening neglected narratives and drawing attention to Other epistemologies. Crossover work considered disruptive in one discipline may find attention in another.

Epistemic disobedience in practice is necessarily political. It challenges the colonial foundations of contemporary meritocracy in global health. It illuminates ideological tensions between fairness and equity. “Fairness” wrongly assumes that levelling the field is enough. “Equity” recognises that players’ histories of relative privilege must be counted for meaningful social justice, retribution, and reparations. Epistemic disobedience demands radical revision of the current order instead of its replication in revised guises. Established ideologies and familiar practices perceived as socially just, simply because they are familiar, unravel under active epistemic disobedience. Dominant views may see these actions as threatening, unfair or wrong. Predictably, resistance and conflict as well as systemic, personal and political attacks and silencing ensue. In practice, epistemic disobedience is not clean, comfortable, quick or quiet. Rather, it does the messy work of undoing colonially founded systems and structures to clear the path for equity-driven, transformative knowledge production.
